# Social Capital and Self-Perceived Quality of Life-Interrelated Predictors of Mediterranean Diet Adherence in Older Adults

**DOI:** 10.3390/nu13093100

**Published:** 2021-09-03

**Authors:** Ioanna Apostolaki, Aleks Pepa, Antonis Vlassopoulos, Maria Kapsokefalou

**Affiliations:** 1 Department of Food Science and Human Nutrition, Agricultural University of Athens, 11855 Athens, Greece; apostola@uoc.gr (I.A.); alekspepa@gmail.com (A.P.); avlassopoulos@aua.gr (A.V.); 2 Department of Social Medicine, Faculty of Medicine, University of Crete, 70013 Heraklion, Greece

**Keywords:** social capital, Mediterranean diet, elderly, mental health, physical health

## Abstract

Living a healthy life in a supporting environment are key elements towards higher diet quality in older age. The Mediterranean Diet in Older Adults (MINOA) study collected cross-sectional data from adults ≥65 years old (*n* = 436) from April 2014 to November 2015 in rural Crete, aiming to understand the interrelations between Mediterranean Diet adherence, Social Capital and Health-Related Quality of Life (HRQL). Multivariate linear regression, carried out using SPSS 20.0, revealed that both Social Capital and HRQL has a positive impact on Mediterranean Diet adherence after adjustment for confounders and independently of each other. Total Social Capital as well as its Value of Life/Social Agency component (β = 0.04 and β = 0.1, *p* < 0.05, respectively) had a positive relationship with Mediterranean Diet adherence. As far as HRQL is concerned, only the Physical Health components were found to have a positive association with Mediterranean Diet adherence (β = 0.09, *p* < 0.001). At the same time Total Social Capital was also seen to have a positive relationship with perceived Physical and Mental Health (β = 0.21 and β = 0.28, *p* < 0.001, respectively). In a population of older adults Social Capital, HRQL and Mediterranean Diet adherence seem to share intricate interrelations that impact both diet quality and quality of life overall.

## 1. Introduction

The global population of adults aged over 65 years old is predicted to reach 1.6 billion individuals by 2050, a 2.6-fold increase compared to 2015 [1]. This shift in demographics challenges the global healthcare system and requires actions to ensure healthy and active ageing [1,2]. Reverting the image of the frail older adult will be at the core of public health with diet being a key modifiable risk factor to be considered [3,4].

Adherence to healthy lifestyles, such as the Mediterranean Diet, has been previously linked to reduced mortality in older adults [5], overall morbidity [6,7], but also to improved cognitive function [7,8,9], reduced risk of dementia [8,9], Alzheimer’s [6,9,10] and Parkinson’s disease [11]. Greater adherence to the Mediterranean Diet was also associated with reduced frailty and Physical Health among community dwelling older adults [12,13,14]. Although research data exist to support a link between higher Mediterranean Diet adherence and better Health Related Quality of Life especially in older adults [15,16,17], data for the opposite association are still scarce [18].

With declining age, adherence to a healthy lifestyle becomes a complex endeavour hindered by multiple factors [19,20]. Other than access and knowledge, social support and the notion of Social Capital is increasingly recognised as an important element in determining the likelihood of adopting a healthy lifestyle in older age [19,21,22,23,24,25]. Social Capital as a notion describes the sense of belonging and being part of a supportive society that shares the same values and creates benefits for the individual and the society as a whole [26]. In its core, Social Capital is split in two main components, the Structural and Cognitive Social Capital, describing the networks, relations and activities that people share, alongside the shared values, perceptions and norms, respectively [26,27]. 

In Greece, and especially in Crete, one of the original locations of the Seven Countries Study, a steady transition towards a more Westernised diet is documented [28,29]. Although such findings are more pronounced in children and adolescents, they are not uncommon between older adults [28,29]. The nutrition transition in Greece started in the 1970s [30] and hence it is safe to speculate that individuals born and raised prior to the nutrition transition would have sufficiently good knowledge of the Mediterranean Diet and so any decline in their adherence would be linked to other social and physical barriers.

**Figure 1:**
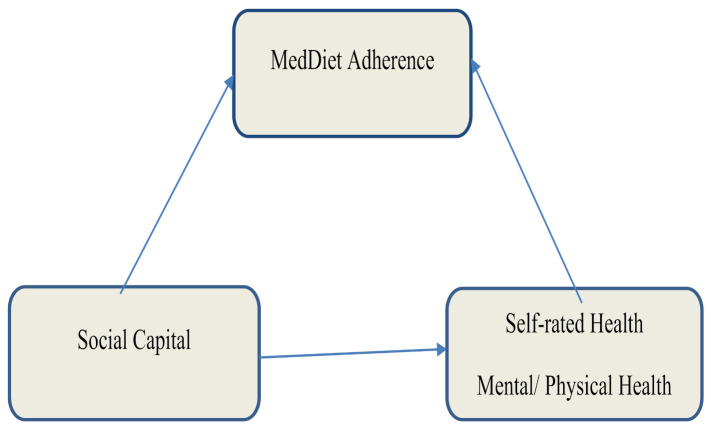
Hypothesised model: research hypothesis of the interactions between Social Capital and Self Rated Health on Mediterranean Diet (MedDiet) adherence.

This study was designed to address this specific gap in the literature by recruiting individuals >65 years old in rural Crete, a demographic born and raised until their early 20s–mid 30s under the Mediterranean Diet as the predominant diet in their region. The aim of the present study was to explore the interrelations among individual Social Capital, health related quality of life, and adherence to the MD in those adults. Our hypothesis is that Med Diet adherence can be positively influenced by Social Capital and self-rated HRQL and that living a healthy life in a supporting environment is an important element of ensuring higher diet quality (Figure 1).

## 2. Materials and Methods

**Figure 2:**
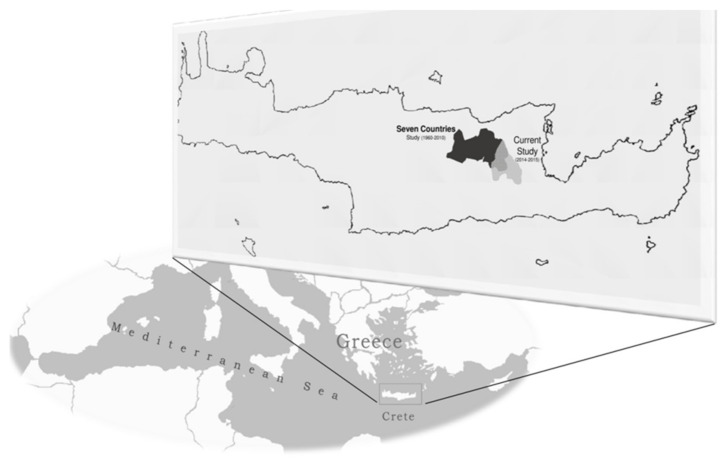
Sampling areas of the current study (MINOA) and the 1960 Cretan cohort of the historical Seven Countries Study (SCS) area.

The MINOA (Mediterranean diet IN Older Adults) is a cross-sectional study conducted between April of 2014 and November of 2015, in the rural area of the Municipality of Minoa, Crete, Greece, one of the regions in the 7 Countries Study (Figure 2). According to the latest census in 2011, 17,563 inhabitants were in the area, of which 5956 (34%) were over 65 years of age. The study protocol was reviewed and approved by the Ethical Committee of the Agricultural University of Athens (181-14/02/2014) and procedures were carried out in accordance with the declaration of Helsinki. All included participants signed a voluntary consent form upon agreeing to participate.

### 2.1. Study Participants

Study participants were community dwelling adults aged ≥65 years old. The MINOA study utilised two recruitment sites in the villages Kastelli and Arkalochori. Participants were recruited from the Open Protection Centers for the Elderly (KAPI), public structures where social workers and health professional and other personnel provide social assistance, first-degree medical care, and recreational activities to the elderly. 

A total of 436 participants (*n* = 251 males, 57.6%; participation rate: 90%) attended an interview with a trained health professional during which self-reported sociodemographic, general health, dietary and anthropometric data were collected. Social Capital and Health–Related Quality of Life (HRQL) data were gathered, using previously validated questionnaires [31,32].

### 2.2. Socio-Demographic, Anthropometric Health Parameters 

Data on age in years, sex and marital status (single, married, divorced or widower) were collected. Self-reported educational level (years spent in school/education) and financial status as mean annual income above or below 10,000 euros were also reported. Living conditions were reported as living alone or with others [33].

Self-reported weight (kg) and height (m) were used to calculate Body Mass Index (BMI) (kg/m^2^) and to assess the presence of overweight/obesity based on established cut-offs (BMI ≥ 25). Participants were also asked to report previous diagnosis of any disease (grouped following ICD-10 categorisation). Participants were grouped as having a diagnosis of 0–2 chronic diseases or above. 

### 2.3. Adherence to the Mediterranean Diet

Mediterranean Diet adherence was measured using a previously validated 11-item food frequency questionnaire [34]. In brief, participants were asked to report the frequency of consumption of 11 main food groups (non-refined cereals and products, potatoes, fruits, vegetables, legumes, fish, red meat and products, poultry, full-fat dairy products, olive oil and alcoholic beverages) in a 5-point scale (never to daily). Increased intake of food groups intrinsic to the Mediterranean Diet received an increasing score, while the opposite was employed for food groups outside of the Mediterranean Diet, resulting in a 55-point aggregate score. Mediterranean Diet adherence was assessed as low, medium and high according to the score’s distribution in tertiles among the study participants [34] 

### 2.4. Social Capital

Individual Social Capital Score was assessed using the Social Capital Questionnaire as translated and validated for Greece [31]. Briefly, participants were asked to indicate their agreement on 36 questions using a 4-point Likert type scale. The 36 questions covered Participation in the Local Community (twelve questions), Feelings of Safety (two questions), Family/Friends Connections (two questions), Value of Life and Social Agency (eleven questions), Tolerance of Diversity (two questions), Work Connections (five questions). In the current study the last module Work Connections was not employed as all participants were retired at the time of the study.

### 2.5. Health-Related Quality of Life

Health-Related Quality Of Life (HRQL) was assessed with the SF-36 questionnaire as performed previously [35] using a version validated for Greece [32]. The SF-36 questionnaire measures perceived quality of life over the past 4 weeks using a series of 36 questions grouped under eight health dimensions: physical functioning (PF, 10 items), physical role functioning (RP, 4 items), bodily pain (BP, 2 items), general health perceptions (GH, 5 items), vitality (VT, 4 items), social functioning (SF, 2 items), emotional role social functioning (RE,3 items) and Mental Health (MH,5 items). Each dimension was scored on a scale from 0 (poor health) to 100 (good health). Physical Functioning (PF), Role-Physical (RP), Bodily Pain (BP) and General Health (GH) were combined to calculate a Physical Component Summary (PCS) score and Vitality (VT), Social Functioning (SF), Role-Emotional (RE) and Mental Health (MH) were used to create the Mental Component Summary (MCS) [36]. 

### 2.6. Statistical Analyses 

Continuous variables were checked for normality using Kolmogorov–Smirnov normality test and were also graphically assessed using P-P plots and histograms. Normally distributed data were expressed as mean with standard deviation (SD), while skewed data as median (Quartile 1–Quartile 3). Categorical values were expressed as percentages (relative frequencies). Differences among groups were tested using ANOVA or the Kruskall–Wallis test for continuous variables and Chi-squared test for categorical variables. Multivariate linear regression was used to study the interrelations between Social Capital, Health-Related Quality of Life (HRQL) and Mediterranean Diet adherence in pairwise associations. The associations were namely the impact of higher Social Capital on Mediterranean Diet adherence, the impact of higher Health Related Quality of Life (HRQL) scores on Mediterranean Diet adherence and the impact of higher Social Capital Scores on HRQL. Multivariate linear regressions were reported in terms of adjusted β coefficients and 95% confidence intervals (linear regression models). Collinearity diagnostics were performed using variance inflation factor (VIF) and tolerance values. Age, gender and having a chronic disease were considered confounders for both Social Capital and HRQL. Marital status and BMI status were confounders for Social Capital, while for HRQL income, educational level, and living conditions were added as additional confounders. Social Capital was also added as a confounder in models with HRQL as the main dependent variable and vice versa. Confounders were selected based on the literature and their correlation with the MedDiet Score (the main dependent variable) in the current population. All statistical analyses were performed using the SPSS 20.0 (SPSS Inc, Chicago, IL, USA) Statistical Package. Significance level was set at 5%.

## 3. Results

The study population was primarily male (58%), married (75.7%) and with a mean age of 75 ± 6.2 years. The average participant was a primary school graduate (6.1 ± 6.2 years spend in school). The majority of the participants earned less than 10,000 annually from pension (73.6%) and had a BMI within the overweight/obesity range (75.7%). Just above half of the participants (57.8%) lived with a previous diagnosis of at least three chronic diseases. The mean Mediterranean Diet Score indicated medium adherence (32 ± 3.6) (Table 1), while the total Social Capital Score was 73.8 ± 8.4 and the PCS score and MCS score for the total sample was 44.4 ± 9.4 and 46.4 ± 9.6, respectively (Table 2).

Men were more likely to report higher adherence to the Mediterranean diet, as were participants who were married at the time of the study and were not living alone. Being diagnosed with three or more chronic diseases was linked to lower likelihood of having high Mediterranean Diet adherence (Table 1).

As far as Social Capital is concerned, higher ratings in the Feeling of Safety/Trust and Value of Life and Social Agency indicators were associated with higher Mediterranean Diet adherence but not the rest of the Social Capital indicators. The contrary was observed for Mediterranean Diet adherence and health, as improved scoring in seven out of eight parameters of the Health-Related Quality of Life (except for the role-emotional health parameter) were associated with higher Mediterranean Diet adherence. There was a stronger positive relationship between Physical Health and Mediterranean Diet adherence than for Mental Health (Table 2). Multivariate linear regression confirms Total Social Capital, Physical Component Summary and Value of Life and Social Agency as independent predictors of Mediterranean Diet adherence (Table 3, Supplementary Table S1). More specifically each point increase in the Total Social Capital was associated with a 0.09-point increase in the Mediterranean Diet Score. Similarly, each point increase in the subcomponent of Value of Life and Social Agency was associated with a 0.10-point increase in Mediterranean Diet Score an impact similar to that of the Physical Component Summary (β = 0.23). 

When the interrelation of Health-Related Quality of Life and Social Capital components were further investigated, a positive relation between Total Social Capital and Physical and Mental Health was observed. However, the effect was twice as strong for Mental Health compared to Physical Health (Table 4). For subcomponents of Social Capital, Value of Life and Social Agency was independently related to improved Physical and Mental Health. On the other hand, for Mental Health only, Participation in the Community and Feeling of Safety/Trust also showed positive effects. In fact, Feeling of Trust/Safety had the strongest relationship with Mental Component Summary compared to all other Social Capital domains. 

## 4. Discussion

This study aims to understand the interconnection of Social Capital, adoption of healthy dietary habits and perceived quality of life in older adults in Crete. Consistent with our research hypothesis, Mediterranean Diet adherence showed a significant positive association with Total Social Capital and especially the “Value of Life and Social Agency” dimension. Perceived Physical Health was also positively related to Mediterranean Diet adherence of this population. Although Mental Health was not on its own related to Mediterranean Diet adherence, it was highly influenced by Social Capital as a whole and specifically the “Participation in the Community”, “Feeling of Safety” and “Value of Life and Social Agency” dimensions. Similar interactions were seen between Social Capital and Physical Health.

We have previously reported gender specific differences in Mediterranean Diet adherence, Social Capital and Perceived Health [37], but the results of this analysis expand these concepts and highlight that those dietary habits are a reflection of social and physical barriers as perceived by older individuals. Previous cross-sectional data in both Mediterranean and non-Mediterranean adult populations confirm that higher Social Capital is often associated with better eating habits and/or perceived quality of life [16,18,38,39]. Focusing on elements of Social Capital has also been suggested as an important driver to improve diet quality, specifically fruit and vegetable intake, even as part of interventional studies [40].

If we focus on life stages during which physical durability is hindered, a better social life in older age was associated with higher MedDiet Scores [33] or other indices of better nutrition [41] but also during pregnancy, higher Mediterranean Diet adherence was linked to higher Social Capital [42]. The observed relationship between Social Capital and diet could be mediated through the sentiment of social trust a significant factor of healthy nutrition but also through interactions that promote the exchange and adoption of healthy behaviours [21]. However, in our study, as it was Value of Life and Social Agency that was directly and independently linked to Mediterranean Diet adherence it is safe to stipulate that psychological element and the sense of community support were the main drivers behind better diets in the current study [43,44]. 

Another important finding was the impact of Physical Health on Mediterranean Diet adherence reported in the current analysis and the lack of an impact for Mental Health. A decline in the ability to ability to shop, carry groceries, grow vegetables or even prepare healthy food without assistance could be detrimental for the adoption or maintenance of healthy diets [45]. However, previous studies have indicated that both physical and Mental Health are associated to healthier diets [17,46]. A potential explanation of the lack of an association between Mental Health and Mediterranean Diet could be the strong interrelationship observed between Social Capital and Mental Health in older adults participating in the MINOA study (Table 4). In the current analysis, almost all elements of Physical Health (Physical Functioning, Role-Physical and General Health) were associated with better Mediterranean Diet adherence (data not shown). The same was not seen for Mental Health for which only Vitality showed a positive relationship with Mediterranean Diet adherence (data not shown). 

As indicated by previous reports, there is a strong positive relationship between Social Capital and quality of life in older adults living in rural areas [45]. Regular access to human contact and social support are associated with better self-assessed Mental and Physical Health [17,47,48,49]. This interaction could be explained if we considered that with the majority of older adults live with some disabilities, from minor kinetic decline to more extensive disabilities. In this context having access to an environment of trust, tolerance, strong connections and a sense of social agency allows the creation of a culture of healthy living but also for the disabilities to be addressed through the support of peer networks [48,49]. In this paradigm, Social Capital and self-perceived quality of life represent who confident a person feels in completing daily tasks on their own (quality of life) and how much peer support is available when needed (Social Capital). In our study, Social Functioning showed a weak association with Mediterranean Diet adherence which might require further investigation as a potential facilitator of better dietary habits in this age group (data not shown).

This study is of particular interest in the study of those two elements (quality of life and Social Capital) on nutrition behaviour in older adults as it studies a unique population. Nutrition behaviour is affected by the degree of knowledge, the skillset and capabilities linked to designing and implementing a healthy lifestyle [19,20]. On that basis, intrinsic (quality of life) and extrinsic (Social Capital) parameters will influence the degree of adherence to a healthy lifestyle [4,7,19,20]. The MINOA study recruited participants from the same location as the Seven Countries Study and from an age group that were alive and even participated in the Seven Countries Study [37]. It is, hence, safe to assume that the participants of the study had a good knowledge and the required skillset to implement a Mediterranean Diet and so the effects of social and personal barriers on nutrition behaviour would be more pronounced and easier to detect. Educational level and financial security have also been shown significantly impact Mediterranean Diet adherence. Previous reports from other Mediterranean countries confirm that although older individuals are more likely to have higher adherence to the Mediterranean Diet the same could be said for those with higher educational status [50,51,52,53]. The same findings are true for the Greek population [28,54]. However, these results may need to be discussed within a different context in the case of older adult populations in the Mediterranean. In Greece, adults aged 55–64 years of age do not attain upper secondary education in their vast majority (75% of the population) [55] and evidence suggest that these numbers might be even larger in adults >65 years old. Moreover, in the majority of the studies mentioned earlier the positive effect of older age was independent of the educational level. That could be explained by the fact that individuals aged >65 years old today have been born and raised with the Mediterranean Diet as the predominant dietary pattern and have experienced a different food environment as the nutrition transition in Greece started during the 70s–80s [30,56]. In fact, high knowledge of dietary pattern as part of the local culture and tradition has been shown to significantly impact dietary habits even during emergency situations as shown during the previous economic crisis and the COVID-19 pandemic [57,58,59]. This suggests that insecurity, financial, food or psychological (due to ageing) could be linked with a tendency to revert to traditional diets.

At the same time, the study is not free of limitations. Although both recruitment locations are rural and fairly isolated communities, participant recruitment through the Open Protection Center for the Elderly (KAPI) may have introduced a selection bias towards individuals with milder disabilities and/or individuals with stronger community ties, as the KAPI structure itself promotes the creation of such networks. The use of self-reported Social Capital and quality of life data could also be mentioned as a limitation, although the methodology used represents standard practice and the questionnaires were validated for the population. The small sample size of the study has also impacted the statistical analysis, as it is possible that specific subdomains of the Social Capital and HRQL scores have higher impacts on Mediterranean Diet adherence or they interact strongly between them to impact dietary behaviour. Such analysis of the possible interactions between scores and subdomains could not be carried out in this analysis but it should be considered for future research. Finally, the cross-sectional nature of the study cannot exclude the possibility of reverse causation and hence stipulations around causality should be done with caution.

## 5. Conclusions

This study highlights the importance of Physical Health and Social Capital, especially Value of Life and Social Agency, as enablers of healthier habits in older age. Social Capital was also an enabler of higher satisfaction with an individual’s quality of life both physical and mental. In a population with high knowledge of the principles and skillset associated to the Mediterranean Diet, Social Capital and perceived health in older age are key determinants of dietary quality.

## Figures and Tables

**Table 1:** Differences in demographic, anthropometric and health related characteristics per Mediterranean Diet adherence level (*n* = 436).

	Total (*n* = 436)	Low ^a^ (*n* = 107)	Medium ^a^ (*n* = 175)	High ^a^ (*n* = 154)	*p* *
Mediterranean Diet Score (0–55)	32(30–35)	28(26–29)	31(30–33)	35(35–37)	0.000
Age (years)	75(70–80)	76(71–80)	74(69–80)	75(70–80)	0.149
Sex (% males)	251(58)	34(31.8)	102(58.3)	115(74.7)	<0.001
Education (years)	6(5–6)	6(4–6)	6(6–7)	6(5–7)	0.011
Marital status (married)	330(75.7)	66(61.7)	140(80)	124(80.5)	<0.001
Financial status (≤10,000)	321(73.6)	89(83.2)	127(72.6)	105(68.2)	<0.05
BMI ≥ 25.0 Kg/m^2^	330(75.7)	79(73.8)	127(72.6)	124(80.5)	0.215
Chronic Diseases ^b^ (3+)	252(57.8)	72(67.3)	104(59.4)	76(49.0)	<0.05
Living alone (% years)	84(19.3)	31(29.0)	26(14.9)	27(17.5)	<0.05

Results are presented as median (Q1–Q3) and n (%). ^a^ Low, middle and high adherence was defined by race—specific Mediterranean Diet Score tertiles: low ≤ 29, middle 30–33, high ≥ 34. ^b^ self-report chronic diseases (hypertension, cardiovascular diseases, respiratory disease, digestive diseases, arthritis–osteoporosis, diabetes mellitus, dyslipidaemias, thyroid diseases). * *p*-values for differences among groups were tested through Kruskal–Wallis test for skewed continuous variables and Chi-square test for categorical variables.

**Table 2:** Differences Social capital and Health Related Quality of Life (HRQL) per Mediterranean Diet adherence level (*n* = 436).

	Total (*n* = 436)	Low ^a^ (*n* = 107)	Medium ^a^ (*n* = 175)	High ^a^ (*n* = 154)	*p* *
Social Capital					
Total Social Capital (range 31–124)	75(68–79.8)	72(66–78)	75(72–80)	75(72–75)	0.082
Participation in the community (range 12–48)	20(18–23)	20(18–22)	21(18–23)	20(18–22.3)	0.632
Feeling of safety/trust (range 2–8)	6(5–7)	6(4–6)	6(5–7)	6(5–7.2)	0.006
Family/friends Connections (range 2–8)	4(3–5)	4(3–5)	4(3–5)	4(3–5)	0.412
Tolerance of Diversity (range 2–8)	4(3–4)	4(3–5)	4(3–4)	4(3–4.2)	0.992
Value of Life and Social Agency (range 11–44)	33(30–35)	32(29–34)	33(30–36)	33(30–36)	0.020
Health Related Quality of Life					
Physical Functioning (PF)	65(40–85)	50(35–70)	65(40–85)	75(50–95)	<0.001
Role-Physical (RP)	62.5(39–81.2)	56.3(25–75)	68.8(43.8–81.3)	65(50–87.5)	0.016
Bodily Pain (BP)	62(42–74)	61(32–72)	62(41–72)	72(52–84)	<0.001
General Health (GH)	50.3 ± 21.9	41.6 ± 21.1	49.3 ± 21.7	57.5 ± 23.4	<0.001
Vitality (VT)	56.2(50–68.8)	56.3(43.8–62.5)	56.3(50–68.8)	62.5(50–75)	<0.001
Social Functioning (SF)	75(62.5–100)	75(50–100)	75(62.5–100)	87.5(75–100)	0.001
Role-Emotional (RE)	58.3(33.3–91.7)	50(25–91.7)	58.3(33.3–91.7)	58(33.3–100)	0.134
Mental Health (MH)	70(55–85)	65(50–80)	75(55–85)	70(65–85)	0.001
Physical Component Summary	44.6(37.3–51.8)	40.7(33.7–48.4)	44.4(37–51.8)	48(40.7–54.3)	<0.001
Mental Component Summary	46.7(39.8–53.8)	44(36.4–52.6)	46.9(39.4–54.4)	47.8 ± 8.6	0.016

Results are presented as median (Q1–Q3) or mean (±sd) and *n* (%).^a^ Low, middle and high adherence was defined by race—specific Mediterranean—and Diet Score tertiles: low ≤ 29, middle 30–33, high ≥ 34. * *p*-values for differences among groups were tested through ANOVA or Kruskal–Wallis test for continuous variables.

**Table 3:** Relationships of Social Capital, Physical Health and Mental Health (SF36) on adherence to Mediterranean Diet using multivariable linear regression models, (*n* = 436).

	Mediterranean Diet Score			
	Crude Coefficients+		Adjusted Coefficients	
	β	95%CIs	β	95%CIs
Social Capital				
Total Social Capital ^a^ (range 31–124)	0.104 *	0.006, 0.08	0.09 *	0.01, 0.08
Participation in the community ^a^ (range 12–48)	0.08	−0.01, 0.17	0.07	−0.02, 0.16
Feeling of safety/trust ^a^ (range 2–8)	0.09	−0.16, 0.39	0.83	−0.02, 0.39
Family/friends Connections ^a^ (range 2–8)	−0.02	−0.35, 0.24	−0.23	−0.37, 0.22
Tolerance of Diversity ^a^ (range 2–8)	0.06	−0.08, 0.38	0.052	−0.10, 0.37
Value of Life and Social Agency ^a^ (range 11–44)	0.11 *	0.03, 0.19	0.10 *	0.01, 0.18
Health Related Quality of Life				
Physical Component Summary, PCS ^b^	0.24 **	0.06, 0.12	0.23 **	0.05, 0.12
Mental Component Summary, MCS ^b^	0.09	−0.002, 0.07	0.02	−0.002, 0.06
Physical Functioning (PF) ^b^	0.21 **	0.02/0.04	0.20 **	0.01/0.04
Role-Physical (RP) ^b^	0.17 **	0.01/0.03	0.15 **	0.01/0.03
Bodily Pain (BP) ^b^	0.15 **	0.01/0.04	0.14 *	0.01/0.04
General Health (GH) ^b^	0.25 **	0.03/0.06	0.26 **	0.03/0.06
Vitality (VT) ^b^	0.20 **	0.02/0.06	0.18 **	0.02/0.06
Social Functioning (SF) ^b^	0.15 **	0.01/0.04	0.14 *	0.01/0.03
Role-Emotional (RE) ^b^	0.06	−0.003/0.02	0.04	−0.01/0.01
Mental Health (MH) ^b^	0.12 *	0.005/0.04	0.11 *	0.004/0.04

Results are presented as β standardised coefficients, 95% confidence intervals. +All crude models were adjusted for gender. ^a^ Adjusted for gender (male/female), age (years), marital status (married/ unmarried, divorced or widower), BMI (<25/≤25) and chronic diseases (0–2/3+). ^b^ Adjusted for gender (male/female), age (years), education (years), financial level (≤10,000/>10,000), living alone (yes/no) and chronic diseases (0–2/3+), Total Social Capital. * *p* < 0.05, ** *p* < 0.001.

**Table 4:** Relationships of Social Capital on Physical Component Summary and Mental Component Summary (SF36), in older adults, aged 65+ years, MINOA study, Crete, Greece (*n* = 436); multivariable linear regression models.

	Physical Component Summary (PCS)				Mental Component Summary (MCS)			
	Crude Coefficients+		Adjusted Coefficients ^a^		Crude Coefficients+		Adjusted Coefficients ^a^	
Social Capital	β	95% CIs	Β	95% CIs	β	95% CIs	β	95% CIs
Total	0.21 **	0.11, 0.32	0.13 *	0.03, 0.23	0.28 **	0.17, 0.38	0.26 **	0.15, 0.37
Participation in the community	0.25 *	0.005, 0.50	0.14	−0.9, 0.37	0.43 *	0.18, 0.68	0.39 *	0.14, 0.64
Feeling of safety/trust	0.58 *	0.29, 1.13	0.41	−0.11, 0.93	1.23 **	0.67, 1.79	1.23 **	0.67, 1.80
Family/friends Connections	0.86 *	0.07, 1.65	0.51	−0.234, 1.26	0.88 *	0.07, 1.69	0.75	−0.07, 1.57
Tolerance of Diversity	0.23	−0.40, 0.86	0.19	−0.40, 0.79	0.71 *	0.07, 1.35	0.70 *	0.06, 1.35
Value of life and Social Agency	0.56 **	0.34, 0.78	0.34 *	0.13, 0.56	0.47 **	0.24, 0.69	0.40 **	0.16, 0.64

* *p* < 0.05, ** *p* < 0.001. Results are presented as β unstandardised coefficients, 95% confidence intervals. + All crude models were adjusted for gender. ^a^ All models adjusted for gender (male/female), age (years), education (years), marital status (married/ unmarried, divorced or widower), financial level (≤10,000/>10,000), BMI (<25/≤25), car (yes/no).

## Supplementary Materials

The following are available online at https://www.mdpi.com/article/10.3390/nu13093100/s1, Table S1: Relationships of Social Capital, Physical Health and Mental Health (SF36) on adherence to Mediterranean diet using multivariable linear regression models (unstandardized coefficients), (*n* = 436).
