# Supplementary material for: Social Capital and Self-Perceived Quality of Life-Interrelated Predictors of Mediterranean Diet Adherence in Older Adults
Source: Nutrients. 2021 Sep 3;13(9):3100. doi: 10.3390/nu13093100 (PMC8465589; doi:10.3390/nu13093100)
Supplement: Supplementary file 1 [file nutrients-13-03100-s001.zip › nutrients-1316860-supplementary.pdf]

Supplementary Table S1. Relationships of Social Capital, Physical Health and Mental Health (SF36) on adherence to Mediterranean diet using multivariable linear regression models (unstandardized coefficients), (n = 436).

	Mediterranean Diet Score			
	Crude Coefficients+		*Adjusted Coefficients*	
	β	**95%CIs**	***β***	**95%CIs**
Total Social Capital [a] (range 31–124)	0.04*	0.006, 0.08	0.04*	0.01, 0.08
Participation in the community [a] (range 12–48)	0.08	-0.01, 0.17	0.07	-0.02, 0.16
Feeling of safety/trust [a] (range 2–8)	0.19	-0.16, 0.39	0.18	-0.02, 0.39
Family/friends Connections [a] (range 2-8)	-0.06	-0.35, 0.24	-0.07	-0.37, 0.22
Tolerance of Diversity [a] (range 2-8)	0.15	-0.08, 0.38	0.13	-0.10, 0.37
Value of Life and Social Agency [a] (range 11–44)	0.11*	0.03, 0.19	0.10*	0.01, 0.18
Physical Health Summary, PCS [b]	0.09**	0.06, 0.12	0.09**	0.05, 0.13
Mental Health Summary, MCS [b]	0.03	-0.002, 0.07	0.03	-0.007, 0.06

Results are presented as β unstandardized coefficients, 95% Confidence Intervals. +All crude models were adjusted for gender. [a] Adjusted for gender(male/female), age (years), marital status (married/ unmarried, divorced or widower), BMI (<25/≤ 25) and chronic diseases (0-2/3+). b Adjusted for gender (male/female), age (years), education (years), financial level (≤10.000/ >10.000), living alone (yes/no) and chronic diseases (0-2/3+), Total Social Capital. *p<0.05, ** p<0.001.
